# Surgical Treatment of Iatrogenic Ventral Glottic Stenosis Using a Mucosal Flap Technique

**DOI:** 10.1111/vsu.12465

**Published:** 2016-03-25

**Authors:** Justine Kane‐Smyth, Timothy P. Barnett, John Mark O'Leary, Padraic M. Dixon

**Affiliations:** ^1^ The Royal (Dick) School of Veterinary StudiesThe University of EdinburghEdinburghUnited Kingdom

## Abstract

**Objective:**

To describe a novel surgical technique for correcting postoperative ventral glottic stenosis (cicatrix or web formation) and the outcome in 2 Thoroughbred racehorses.

**Study Design:**

Retrospective case report.

**Animals:**

Thoroughbreds diagnosed with ventral glottic stenosis (n=2).

**Methods:**

Horses presenting with iatrogenic ventral glottic stenosis and resultant exercise intolerance and abnormal exercise‐related noise were anesthetized and a midline sagittal skin incision was made over the ventral larynx and between the sternohyoideus muscles overlying the cricothyroid notch. The cricothyroid ligament, attached laryngeal cicatrix, and overlying mucosa were sagittally sectioned at the dorsal aspect of the cicatrix on the left side. The laryngeal mucosa, cicatrix, and underlying cricothyroid ligament immediately rostral and caudal to the cicatrix were sectioned in a medial (axial) direction as far as the right side of the cricothyroid notch. After resection of the majority of the attached cicatrix tissue, the residual mucosal flap (attached to the right side of the larynx) was reflected ventrally and sutured to the attachment of the cricothyroid ligament on the right side of the cricothyroid notch, creating an intact mucosal layer on the right side of the ventral larynx.

**Results:**

Both horses had good intralaryngeal wound healing with minimal redevelopment of ventral glottic stenosis at 5 and 9 months postoperatively and were successfully returned to racing with complete absence of abnormal respiratory noise.

**Conclusion:**

The unique laryngeal anatomy of horses, with a cartilage‐free ventral laryngeal area (cricothyroid notch), allowed the use of this novel surgical technique to successfully treat ventral glottic stenosis.

Laryngeal stenosis (cicatrix or webbing), usually involving the rima glottidis (ie, glottic stenosis), is a rarely reported disorder in horses. It is characterized by the presence of a mucosa‐covered, fibrous band of tissue stretching across the laryngeal lumen, usually on its ventral aspect and less commonly dorsally.^1^, ^2^ With the exception of a single report of congenital glottic stenosis associated with laryngeal dysplasia,^3^ glottic stenosis in horses is typically iatrogenic, usually after bilateral laryngeal surgery,^1^, ^2^, ^4^, ^5^ or rarely after severe external laryngeal trauma.^1^ In certain geographical regions, glottic stenosis can occur as part the nasopharyngeal cicatrix syndrome.^6^ Any endolaryngeal insult that causes mucosal ulceration can result in intraluminal protrusion of granulation tissue. With extensive or multiple mucosal defects, protruding laryngeal granulation tissue on one side of the larynx may adjoin granulation tissue on the opposite side, ultimately leading to a band of mature fibrous tissue spanning the laryngeal lumen that later becomes epithelialized.^7^

Unilateral left vocalcordectomy or ventriculocordectomy were traditional treatments for horses with recurrent laryngeal neuropathy. However, with the advent of dynamic respiratory endoscopy, it became apparent that concurrent dynamic right vocal cord instability was present in some cases and consequently, bilateral laryngeal surgery has been performed in some cases.^8^, ^9^, ^10^ Stenosis has not been reported after bilateral ventriculectomy, possibly because intact mucosa remains on the medial aspect of both vocal cords after this surgery. In contrast, bilateral vocalcordectomy or ventriculocordectomy leaves areas denuded of epithelium on opposite sides of the ventral larynx, which can potentially result in cicatrix formation.^1^ Transendoscopic laser surgery is widely used for equine vocalcordectomy and ventriculocordectomy, including unilateral and bilateral vocalcordectomy in both experimental and clinical cases.^9^, ^11^, ^12^, ^13^, ^14^, ^15^ There are few reports of iatrogenic cicatrix formation after laser laryngeal surgery^5^ and it is unclear whether this disorder is under‐reported.

Glottic stenosis is well described in other species, including people, where 40% of cases are congenital.^16^ Acquired glottic stenosis in people is most commonly caused by prolonged endotracheal intubation, with intubation of 10 or more days is reportedly associated with a 14% prevalence of laryngotracheal stenosis.^7^ Thermal or chemical intraluminal injury from ingested fluids, laryngeal surgery, or severe external laryngeal trauma, such as knife or bullet wounds, are less common causes of acquired glottic stenosis in people.^17^ Canine glottic stenosis is usually a sequel to bilateral vocalcordectomy per os, especially when these procedures are performed without primary closure of the mucosal defect.^18^, ^19^ The reported treatments for glottic stenosis in horses include surgical resection of the stenosis, reported to be unsuccessful, or long‐term placement of an intralaryngeal stent.^1^ The purpose of this report is to describe a new surgical technique that was used to correct iatrogenic ventral glottic stenosis in 2 horses.

## Clinical Report

### Case 1

**Figure 1:**
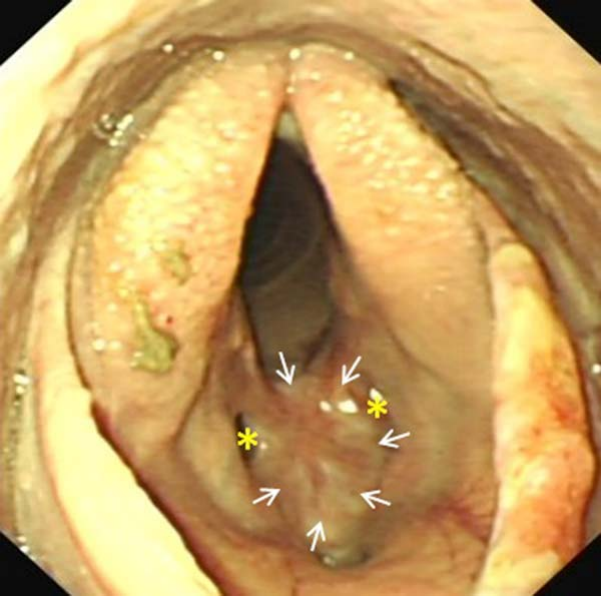
Case 1: Preoperative endoscopic image illustrating the ventral laryngeal cicatrix (arrows) that extended approximately halfway up the vocal cords and extended rostrally and caudally on the laryngeal floor. Residual small lateral ventricles (asterisks) are present. The soft palate is transiently displaced and food particles are present on the surfaces of the arytenoids.

A 2‐year‐old Thoroughbred racing gelding had been successfully treated for epiglottic entrapment 17 weeks before presentation. Exercise intolerance and respiratory noise had subsequently remained during galloping, however. Six weeks after epiglottic surgery, overground exercising endoscopy demonstrated bilateral vocal cord and aryepiglottic fold collapse. Bilateral vocal cord and bilateral partial aryepiglottic fold resections were performed using a diode laser (unknown Joules of energy used). Postoperative topical corticosteroid and parenteral flunixin meglumine were administered. Upper respiratory endoscopy was performed 6 weeks after laser surgery to investigate respiratory noise audible during trotting exercise, and revealed abnormal laryngeal scarring. Five weeks later, examination at our clinic confirmed the presence of a marked ventral glottic stenosis caused by cicatrix formation between the 2 vocalcordectomy sites (Fig 1). The ventrally located web measured ∼30 mm rostrocaudally and 25 mm dorsoventrally. Only residual small lateral ventricles were present, suggesting that collateral thermal damage to the lateral ventricular mucosa at the time of vocalcordectomy had also caused occlusion of the ventral aspects of the lumina of the ventricles. The aryepiglottic fold resection sites had fully healed and left‐sided, resting laryngeal function was graded Havemeyer grade III.1.^20^

**Figure 2:**
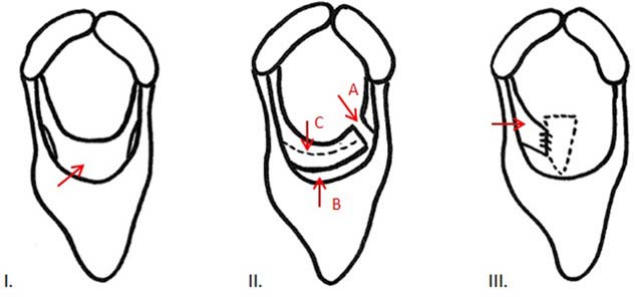
Schematic diagrams illustrating the surgical procedure. I: Rostrocaudal view illustrating the formed cicatrix (arrow). II: Illustrating the 3 incisions: (A) rostro‐caudal incision at the left dorsal aspect of the cicatrix; (B) left to right transverse incision rostral to the cicatrix; and (C) left to right transverse incision immediately caudal to the cicatrix. III: Illustrating the mucosal flap (arrow) sutured to the right side of the open cricothyroid notch (hatched line).

**Figure 3:**
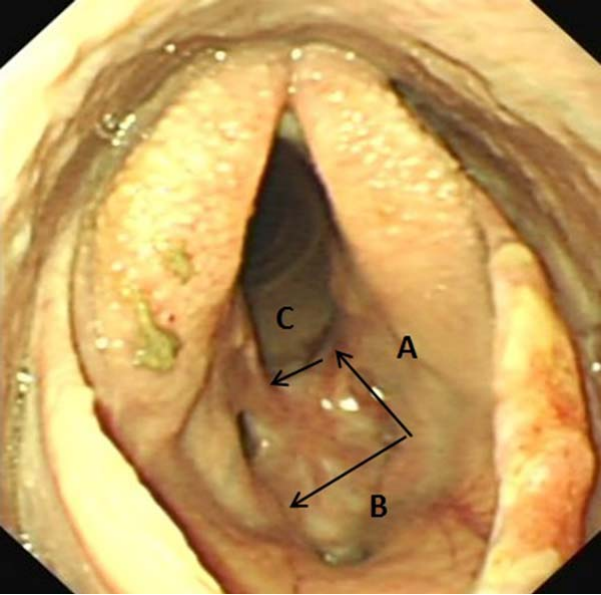
Case 1: Endoscopic image of the cicatrix with arrows demonstrating the site and direction of the 3 laryngeal incisions. First, a rostrocaudal incision at the left dorsal aspect of the cicatrix (A); second, a left to right transverse incision rostral to the cicatrix (B); and third, a left to right transverse incision immediately caudal to the cicatrix (C).

**Figure 4:**
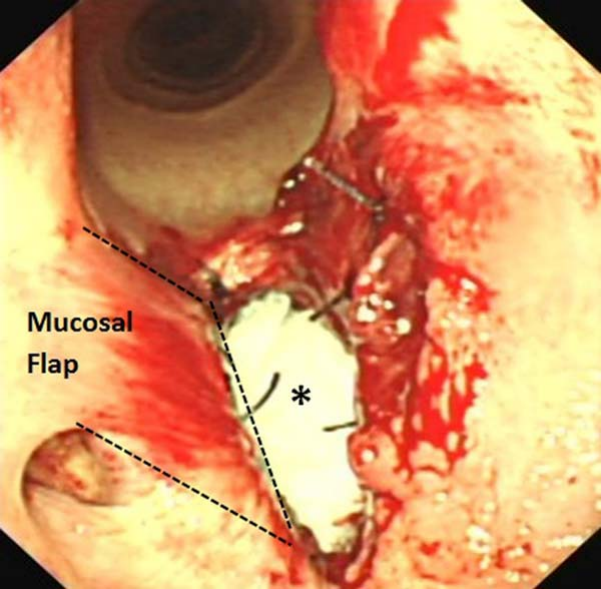
Case 1: Intraoperative endoscopic image showing the ventrally hinged, right‐sided laryngeal mucosal flap (outlined) that has been sutured to the right side of the cricothyroid notch. The incised edge of the laryngotomy incision is still open and the asterisk indicates the open cricothyroid notch. The residual left lateral ventricle has been removed and its ostium sutured closed. Loose mucosa above the horizontal incision on the left side has been sutured to adjacent submucosal tissue.

After anesthetic induction, the glottis was too small to accommodate a 24 mm diameter endotracheal tube (for inhalation anesthesia during surgical site preparation). Total intravenous anesthesia was employed throughout the surgical procedure using a constant rate infusion of midazolam (0.05 mg/mL), ketamine (1.3 mg/mL), and detomidine hydrochloride (0.02 mg/mL). Using a ventral midline approach, the skin over the larynx was incised and the sternohyoideus muscles were bluntly separated to expose the cricothyroid ligament, much of which was embedded in the ventral aspect of the fibrous cicatrix. Under nasopharyngeal endoscopic guidance, the dorsal limit of the left side of the cicatrix was determined by inserting hypodermic needles (21 gauge, 3.75 cm long) through the cricothyroid ligament and cicatrix into the laryngeal lumen. A sagittally orientated, ∼30 mm long, paramedian laryngotomy incision was then made over the left dorsal aspect of the cicatrix, just ventral to the left arytenoid vocal process. Using a #10 scalpel blade, 2 transverse incisions were then made from left to right, in the laryngeal mucosa and underlying cricothyroid ligament, rostral and caudal to the cicatrix. This created an ∼30 mm wide mucosa‐covered fibrous flap attached to the right laryngeal wall and hinged just below the right arytenoid. (Figs 2 and 3). Most of the attached scar tissue and the residual cricothyroid ligament were resected, leaving just a few millimeters of fibrous tissue remaining beneath the intact laryngeal mucosa. Some scar tissue at the right dorsal aspect of the cicatrix was submucosally resected. The distal free aspect of this mucosal flap was then stretched and sutured to the attachment of the cricothyroid ligament on the right side of the cricothyroid notch using simple interrupted sutures of 2‐0 polyglactin 910 (Figs 2 and 4). Some loose laryngeal mucosa dorsal to the cicatrix resection site on the left side was sutured to the submucosa. The residual left lateral ventricular mucosa from the original laryngeal laser surgery was then excised and its ostium was sutured closed. As no identifiable cricothyroid ligament remained, the cricothyroid notch was left open (Fig 4) but the overlying sternohyoideus muscles were reapposed using 0 polyglyclactin 910 in a simple continuous pattern, and the skin was closed primarily using 0 polyglactin 910 in a simple interrupted pattern.

**Figure 5:**
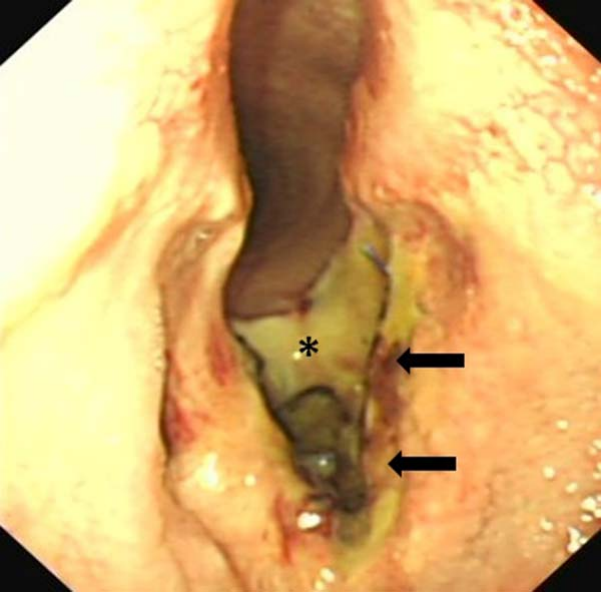
Case 1: Endoscopic image of the surgical site 24 hours after cicatrix resection showing swelling of the right‐sided mucosal flap. The caudal aspect of the exposed cricothyroid notch is covered in fibrin (asterisk) as is the left ventral aspect of the glottis (arrows).

**Figure 6:**
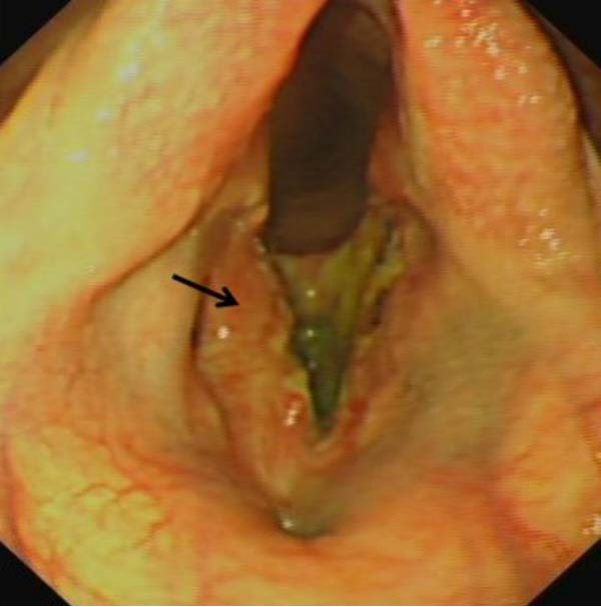
Case 1: Endoscopic image of the surgical site 5 days after cicatrix resection showing more marked swelling of the right mucosal flap (arrow).

Flunixin meglumine (1.1 mg/kg IV) was administered preoperatively and continued twice daily for 48 hours postoperatively. Neomycin (5 mg/kg IM) and procaine penicillin (25,000 IU/kg IM) were administered preoperatively and continued twice daily for 4 days followed by a 7 day course of trimethoprim/sulfadiazine (30 mg/kg orally twice daily). Endoscopic assessment was performed daily for 5 days postoperatively and showed the repositioned mucosal flap to be inflamed, but intact (Figs 5 and 6).

**Figure 7:**
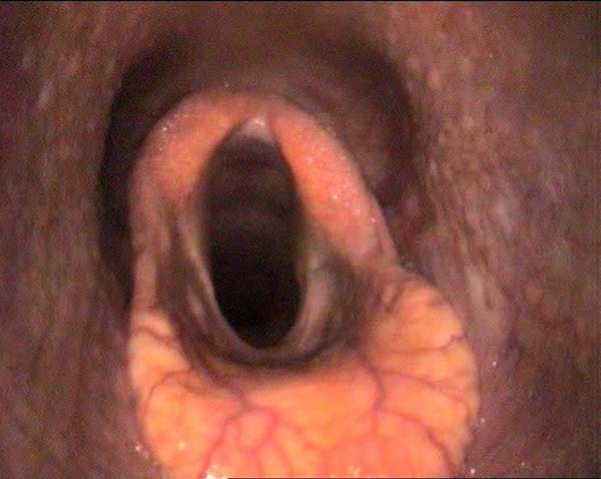
Case 1: Resting endoscopy image 5 months after laryngeal cicatrix resection. There is some residual thickening of the left ventral aspect of the left and right sides of larynx and also 5 to 10 mm deep generalized fibrosis of the laryngeal floor. Left laryngeal hemiparesis (Havemeyer III:1) is still present.

Six weeks after corrective surgery, the horse returned to training with no abnormal exercise‐related noise reported and returned to competitive racing 16 weeks after surgery. The horse raced successfully 11 times that season with complete absence of abnormal exercise‐related respiratory noises. Resting endoscopy 5 months postoperatively revealed a marked improvement in glottic diameter with a slight reduction in the laryngeal lumen due to of the presence of a ∼5 mm deep, generalized fibrosis of the ventral laryngeal floor (Fig 7). Resting laryngeal function remained unchanged (Havemeyer III.1).

### Case 2

**Figure 8:**
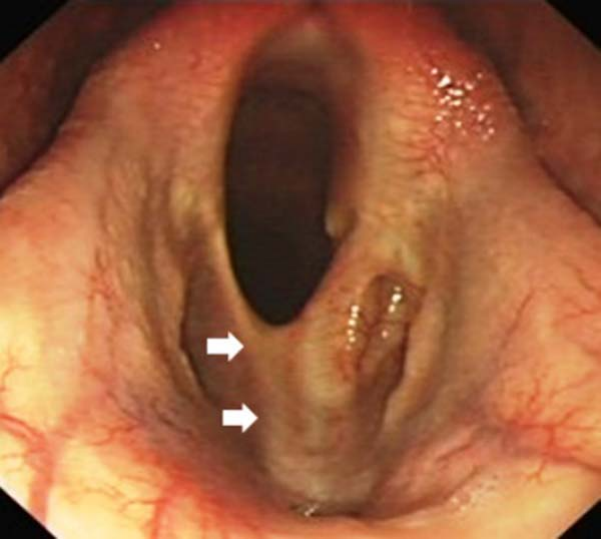
Case 2: Preoperative endoscopic image showing a marked ventral glottic stenosis. The outline of the intact right vocal cord can be seen (arrows) with the cicatrix attached to its ventromedial aspect. The ostium of the right lateral ventricle is pulled open by the cicatrix and the dorsal aspect of the left lateral ventricle is also patent.

Overground endoscopy of a 5‐year‐old female Thoroughbred racehorse with upper respiratory noise during exercise showed dorsal displacement of the soft palate (DDSP) and medial prolapse of the left vocal cord. A laryngeal tie‐forward procedure^21^ using braided polyblend sutures (Arthrex GmbH, Karlsfield, Germany) and a left‐sided diode laser vocalcordectomy (unknown Joules of energy used) were subsequently performed. The details of postoperative medications were unobtainable. The mare was rested at pasture and received no veterinary attention until 12 weeks after surgery, when a small draining tract was found at the caudal aspect of the tie‐forward incision site, on ventral midline at the level of the rostral larynx. Resting upper respiratory endoscopy at that time showed apparent intralaryngeal scarring. After referral to our clinic 1 week later, endoscopic examination showed ventral glottic stenosis (Fig 8) and ultrasonography identified a small anechoic pocket, suggestive of abscess formation, adjacent to where the tie‐forward prosthesis passed through the right side of the thyroid cartilage.

**Figure 9:**
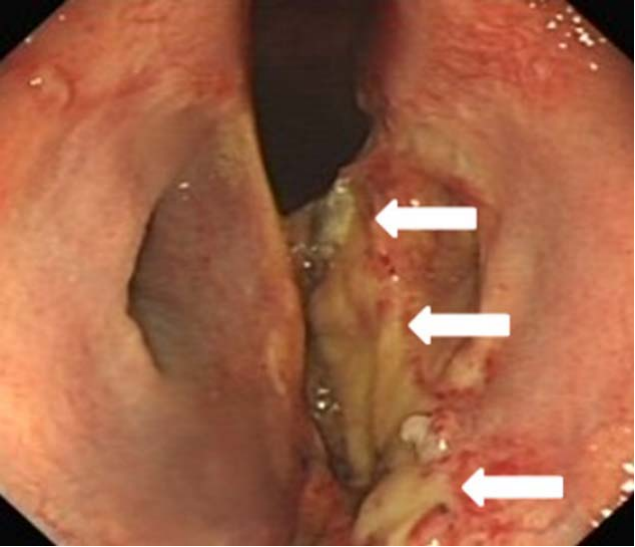
Case 1: Endoscopic image of the surgical site 24 hours postoperatively, showing swelling of the right‐sided mucosal flap and a large mucosal defect on the left ventral aspect of laryngeal lumen (arrows).

After induction of general anesthesia, it was only possible to insert a 22 mm diameter endotracheal tube into the trachea. Dissection of the sinus tract on ventral midline, overlying the rostral aspect of the thyroid cartilage revealed communication with an infected tie‐forward prosthesis adjacent to the right thyroid cartilage, with both prostheses embedded in thick fibrous tissue. Both prostheses were fully removed, a Penrose drain (10 mm diameter) was inserted and the wound was partially sutured closed using 0 polyglactin 910. The horse was then extubated and the remaining surgery performed under intravenous anesthesia using the same protocol as described above. Caudal to the incision used to remove the prostheses, a midline laryngotomy incision was made through the skin and between the sternohyoideus muscles until the fibrotic cricothyroid ligament was reached, as described for Case 1. The left dorsal limit of the cicatrix on the left side was determined again using hypodermic needles (Fig 9). Scar tissue was dissected from the cicatrix using a #10 scalpel blade. The cicatrix was sagittally transected above its left dorsal limit, ventral to the left corniculate process. Two transverse incisions from left to right were then made, rostral and caudal to the cicatrix, creating a mucosa covered fibrous flap, hinged below the right vocal process, as described for Case 1 above (Fig 2). After resection of most of the remaining cicatrix scar tissue, the intact mucosal flap was retracted ventrally and sutured to the attachment of the cricothyroid ligament on the right side of the cricothyroid notch using 2‐0 polyglactin 910. The caudal aspect of the cricothyroid ligament was not attached to the cicatrix in this case. The incision was partially closed using 2‐0 polyglactin 910 in a simple interrupted pattern. The sternohyoideus muscles were reapposed, and the skin over the laryngotomy was closed primarily as in Case 1.

The horse was administered cefquinome (1 mg/kg IV twice daily) and flunixin meglumine (1.1 mg/kg IV twice daily for 4 days), followed by a 7‐day course of trimethoprim/sulfadiazine (30 mg/kg orally twice daily) and phenylbutazone (2.2 mg/kg orally twice daily). Endoscopic reevaluation was performed on 3 occasions in the first week postoperatively and showed local mucosal inflammation, with the surgical correction appearing intact (Fig 9).

**Figure 10:**
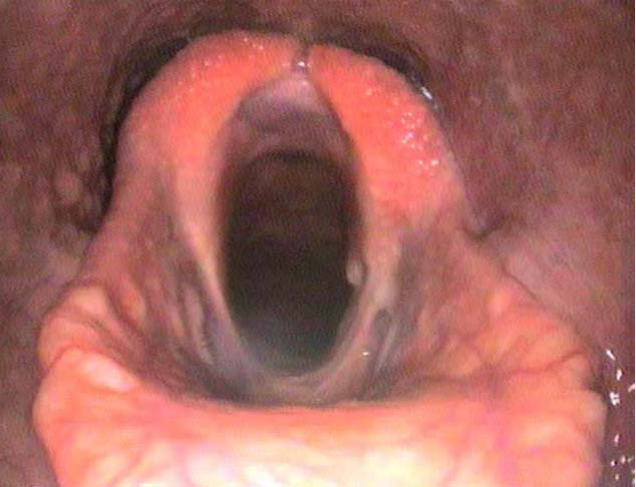
Case 2: Resting endoscopy 9 months after resection of ventral laryngeal cicatrix. There is slight thickening bilaterally at the surgical sites in the ventral larynx and a slight generalized thickening of the laryngeal floor.

The mare returned to training 8 weeks postoperatively, with no reported abnormal exercise‐related noise, and raced 12 weeks later. In total, she raced 5 times that season with complete absence of abnormal exercise‐related respiratory noise. Resting endoscopy 9 months after cicatrix resection showed minimal residual scarring at the ventrolateral aspects of the larynx; however, diffuse generalized scarring was present on the laryngeal floor (Fig 10).

## Discussion

In this report, we describe the successful use of a novel surgical technique to correct iatrogenic ventral glottic stenosis that developed after laryngeal laser surgery in 2 horses. The risk of development of glottic stenosis after simultaneous bilateral vocalcordectomy was made known to the owner in Case 1. The preferred option of performing staged vocalcordectomy procedures some weeks apart was apparently declined for economic reasons. Despite reportedly leaving the base of the vocal cords intact and administering postoperative topical steroid and systemic nonsteroidal anti‐inflammatory therapy, laryngeal cicatrix formation occurred, as has previously been reported after bilateral surgical vocalcordectomy.^4^ It is probable that inadvertent thermal damage to the bases of the resected vocal cords and the intervening mucosa on the floor of the larynx occurred in this horse. Because of this unavoidable risk, concurrent bilateral laser vocalcordectomy should be avoided.

The development of glottic stenosis in Case 2 after unilateral vocalcordectomy was considered unusual. The medial border of an otherwise intact right vocal cord was attached by a cicatrix to the left vocalcordectomy site. No evidence of laser thermal damage was reportedly present on the right vocal cord after the left vocalcordectomy. However, the later presence of cicatrix at this site suggests that the mucosa of the ventromedial edge of the right vocal cord may have been inadvertently thermally injured, with delayed clinical onset of mucosal damage. The infection at the right thyroid cartilage, associated with the tie‐forward procedure, was distant to the glottic stenosis and these two disorders appeared to be unrelated. A similar case that developed ventral glottic stenosis after unilateral laser vocalcordectomy using an unknown laser type and with inadvertent damage to the opposite vocal cord has been previously reported.^5^ In support of the hypothesis that cicatrix formation in Case 2 was related to collateral laser thermal injury, Parente reported that adjacent tissues which appear normal after laser surgery but may later undergo necrosis because of inadvertent transmission of excessive thermal energy.^22^

The duration of laser vocalcordectomy procedures can vary greatly depending on operator skill, anatomical differences between cases, and the occurrence and degree of surgical hemorrhage from vocal cord vasculature. Obviously, cases that receive the highest doses of laser thermal energy administered to the laryngeal tissues are at highest risk of developing collateral thermal damage. While using a diode laser at 20 watts and administering a mean of 8500 Joules for experimental equine ventriculocordectomy, no damage was grossly or histologically found on the opposite vocal cord.^23^ In our report, the total laser energy doses administered to either of the horses in the initial surgeries was unknown. Ahern and Parente noted that prevention of laser damage during equine laryngeal surgery should be the surgeon's aim, by using the minimum amount of energy to complete the procedure and keeping the tissue under tension during resection.^24^

Ultrasonography was performed in Case 2 to assess the draining tract associated with the tie‐forward incision. In retrospect, preoperative ultrasonographic assessment of the dimensions and site of the cicatrix would have been useful in surgical planning. Computed tomographic and magnetic resonance laryngeal imaging have been utilized to evaluate laryngeal cicatrices in people, both to provide information on cicatrix dimensions and also identify involvement of adjacent structures.^25^ Other reported techniques for treating equine glottic stenosis include surgical excision of the cicatrix, which may ultimately result in a wider and taller cicatrix.^1^, ^5^ Alternatively, the use of an intralaryngeal circular stent sutured over the site of the resected cicatrix has been successful, but requires long‐term hospitalization and monitoring.^1^ Other potential treatment options include free oral mucosal grafts, as performed in people^26^ or possibly a pedicle graft of the very mobile equine aryepiglottic mucosa. Such intralaryngeal surgery would be technically difficult to perform with the horse in dorsal recumbency, through the narrow cricothyroid space. In a recent report, the most common treatment of glottic stenosis in people was endoscopic CO_2_ laser surgery.^27^ Greater precision and reduced collateral thermal damage is achievable using CO_2_ lasers compared with diode lasers (known to have been used in Case 2). In the current cases, however, the cicatrices were tall dorsoventrally and deep rostrocaudally, and it was therefore believed that attempted laser resection, even using a CO_2_ laser, would very likely have caused even more extensive laryngeal scarring.

Infection of equine tie‐forward surgical sites, containing a braided nonabsorbable prosthesis, is reported in 1% (NG Ducharme personal communication) to 4% of cases.^28^ The cause of infection in Case 2 was unknown, although it has been suggested that surgical site contamination may occur during intraoperative head flexion when the prosthesis is being tightened. Alternatively, infection may have been related to performing a simultaneous ventriculocordectomy. The tie‐forward prostheses are separated from the laryngeal lumen by only the cricoarytenoideus lateralis and are therefore, in very close proximity to the ventriculocordectomy site. As the ventriculocordectomy site is left open to heal by second intention, this may provide an entry route for bacterial contamination of the prostheses. It may be prudent to avoid performing the two procedures simultaneously. Because the tie‐forward prosthesis removal was performed 13 weeks after its insertion, it was believed that the typically marked fibrosis present would maintain the larynx in a more rostral position after surgery. The horse raced without abnormal respiratory noise after prosthesis removal along with laryngeal cicatrix surgery, indicating that clinically detectable DDSP did not recur during this time.

The authors routinely perform primary closure of the cricothyroid ligament and partial closure of the sternohyoideus muscles and skin after conventional laryngotomy. Previously, it has been reported that such wound closure results in lower rates of incisional complications compared with leaving the incision open to heal by second intention.^29^, ^30^ It was only possible to reappose the muscle and skin in Case 1; however, the incision reportedly healed without complication.

The unique laryngeal anatomy of the horse, with cartilage‐free ventral laryngeal area (cricothyroid notch), allowed the described novel surgical technique to be used successfully to correct ventral glottic stenosis. The submucosal resection of most of the cicatrix followed by use of a unilateral mucosal flap technique allowed good laryngeal wound healing with minimal redevelopment of stenosis.

## Disclosure

The authors declare no conflicts of interest related to this report.
